# Anti-Retroviral Lectins Have Modest Effects on Adherence of *Trichomonas vaginalis* to Epithelial Cells *In Vitro* and on Recovery of *Tritrichomonas foetus* in a Mouse Vaginal Model

**DOI:** 10.1371/journal.pone.0135340

**Published:** 2015-08-07

**Authors:** Aparajita Chatterjee, Daniel M. Ratner, Christopher M. Ryan, Patricia J. Johnson, Barry R. O’Keefe, W. Evan Secor, Deborah J. Anderson, Phillips W. Robbins, John Samuelson

**Affiliations:** 1 Department of Molecular and Cell Biology, Boston University Goldman School of Dental Medicine, Boston, Massachusetts, United States of America; 2 Department of Microbiology, Immunology, and Molecular Genetics, David Geffen School of Medicine, University of California Los Angeles, Los Angeles, California, United States of America; 3 Molecular Targets Laboratory, Center for Cancer Research, Frederick National Laboratory for Cancer Research, Frederick, Maryland, United States of America; 4 Division of Parasitic Diseases and Malaria, Centers for Disease Control and Prevention, Atlanta, Georgia, United States of America; 5 Department of Obstetrics and Gynecology, Boston Medical Center, Boston, Massachusetts, United States of America; Aga Khan University Hospital Nairobi, KENYA

## Abstract

*Trichomonas vaginalis* causes vaginitis and increases the risk of HIV transmission by heterosexual sex, while *Tritrichomonas foetus* causes premature abortion in cattle. Our goals were to determine the effects, if any, of anti-retroviral lectins, which are designed to prevent heterosexual transmission of HIV, on adherence of *Trichomonas* to ectocervical cells and on *Tritrichomonas* infections in a mouse model. We show that *Trichomonas* Asn-linked glycans (*N*-glycans), like those of HIV, bind the mannose-binding lectin (MBL) that is part of the innate immune system. *N*-glycans of *Trichomonas* and *Tritrichomonas* bind anti-retroviral lectins (cyanovirin-N and griffithsin) and the 2G12 monoclonal antibody, each of which binds HIV *N*-glycans. Binding of cyanovirin-N appears to be independent of susceptibility to metronidazole, the major drug used to treat *Trichomonas*. Anti-retroviral lectins, MBL, and galectin-1 cause *Trichomonas* to self-aggregate and precipitate. The anti-retroviral lectins also increase adherence of ricin-resistant mutants, which are less adherent than parent cells, to ectocervical cell monolayers and to organotypic EpiVaginal tissue cells. Topical application of either anti-retroviral lectins or yeast *N*-glycans decreases by 40 to 70% the recovery of *Tritrichomonas* from the mouse vagina. These results, which are explained by a few simple models, suggest that the anti-retroviral lectins have a modest potential for preventing or treating human infections with *Trichomonas*.

## Introduction

*Trichomonas vaginalis* is a widely prevalent sexually transmitted human parasite, while *Tritrichomonas foetus* (used in a mouse model of *Trichomonas* infection) is a widely prevalent sexually transmitted parasite of cattle [1–3]. While there is a second species that colonizes the upper digestive track of birds (*Trichomonas gallinae*) and growing evidence for a two-type structure to clinical isolates of *Trichomonas vaginalis*, we will use “*Trichomonas*” to refer to lab strains and clinical isolates studied here [4]. *Trichomonas*, which infects nearly four million women in the US each year, causes vaginitis, increases the risk of miscarriage, and increases rates of transmission of HIV from men to women [5–8]. *Trichomonas* infections are treated by systemic administration of metronidazole (also known as Flagyl) or tinidazole (Tindamax). However, ~4% of clinical isolates of *Trichomonas* show reduced *in vitro* susceptibility to metronidazole, and adequate safety data are not available regarding the use of tinidazole in pregnancy [9]. While there is no vaccine against *Trichomonas*, numerous candidate antigens have been identified (reviewed in [10]). Although there is no topical reagent approved to reduce the likelihood of *Trichomonas* infection, anti-poly-N-acetyl-glucosamine (PNAG) antibodies and *Sapindus* saponins have been proposed [11,12].

*Trichomonas* causes vaginitis when it penetrates the mucus layer, binds to and lyses ectocervical cells, and migrates across host tissues [13,14]. *Trichomonas* changes from a flagellated to an ameboid form, which is associated with changes in gene expression, actin polymerization, and release of exosomes that fuse with host cells and cause the release of cytokines [15–18]. Adherence to and lysis of a benign prostatic hyperplasia cell line by *Trichomonas* are by similar mechanisms, while a *Trichomonas* homolog of macrophage inhibition factor may cause prostatic cell growth and inflammation [19].

As has been shown for bacteria and other parasites, sugars on the surface of *Trichomonas* and host lectins that recognize these sugars are involved in adhesion and pathogenesis [20,21]. Adhesion of *Trichomonas* to human ectocervical cells *in vitro* is inhibited by parasite lipoglycan (LG) that contains chains *N*-acetyllactosamine (LacNAc), which is galactose linked ß1-4 to *N*-acetylglucosamine (GlcNAc). LacNAc is also bound by galectin-1, a component of the innate immune response that is present in serum, in vaginal secretions, and on the surface of vaginal epithelial cells [22,23]. Mutants of *Trichomonas* that do not bind ricin B chain (i.e. are ricin-resistant) have decreased LacNAc in their LG, show decreased adherence to and lysis of ectocervical cells *in vitro*, and show decreased binding of host galectin-1 [24]. In addition, the plant lectin Concanavalin A inhibits adherence of *Trichomonas* to cell culture monolayers [25].

We have made a number of basic observations concerning the Asn-linked glycans (*N*-glycans) of *Trichomonas*. The parasite *N*-glycan precursor contains seven sugars (Man_5_GlcNAc_2_-PP-dolichol) rather than the 14-sugar precursor (Glc_3_Man_9_GlcNAc_2_-PP-dolichol) made by most animals, plants, and fungi [26]. The *Trichomonas N*-glycan is glucosylated by a uridine diphosphate glucose (UDP-Glc) glucosyltransferase that is part of the *N*-glycan-dependent quality control (NG-QC) of glycoprotein folding [27]. Conversely, the *Trichomonas N*-glycan is trimmed by an ER mannosidase that resembles those involved in *N*-glycan-dependent ER-associated degradation (NG-ERAD) of misfolded glycoproteins. There is positive selection for sites of *N*-linked glycosylation in secreted and membrane proteins of *Trichomonas* and other eukaryotes with NG-QC [28].

*Trichomonas* glycoproteins that contain *N*-glycans or *N*-glycan sites, which were identified by mass spectrometry, include important virulence factors shared with *Tritrichomonas*, as well as many unique proteins that appear to be absent from the predicted proteins of *Tritrichomonas* (our unpublished data and [29]). Shared virulence factors suggest *Tritrichomonas*, which causes a robust vaginal infection in mice that ascends into the uterus, may be a good surrogate for *Trichomonas* [2]. In contrast, *Trichomonas* causes transient and weak mouse infections that depend upon inducing estrus, treatment with dexamethasone, and co-infection with *Lactobacilli* [3].

Many *Trichomonas N*-glycans are not processed in the ER or Golgi and so have a single mannose arm that is similar to the three mannose arms present on unprocessed *N*-glycans on gp120 of HIV [26,30,31]. In contrast, most human *N*-glycans are extensively processed in the ER and Golgi and contain LacNAc arms capped by sialic acid, as well as fucose attached to GlcNAc at the base [32]. The unprocessed *N*-glycans of HIV bind mannose-binding proteins of the innate immune response, which include the mannose-binding lectin (MBL) in serum and vaginal secretions, the mannose receptor on macrophages, and DC-SIGN on dendritic cells [33–36]. MBL, which has a carbohydrate recognition domain and a collagenous domain, forms oligomers that activate complement by the lectin-mediated pathway. The unprocessed *N*-glycans of HIV are also the target of a broadly neutralizing monoclonal antibody (2G12) derived from a long-term non-progressor infected with HIV, as well as anti-retroviral lectins (cyanovirin-N from a cyanobacterium and griffithsin from a red alga) developed as topical therapeutics to prevent the heterosexual spread of HIV [37–40]. These anti-retroviral lectins are also active against herpes simplex virus (HSV), hepatitis C virus (HCV), and influenza virus [41–43].

The first goal of our studies was to explore the role, if any, of *Trichomonas N*-glycans on pathogenesis. To do this, we determined whether recombinant human MBL labels the surface of *Trichomonas*. The specificity of MBL for *Trichomonas N*-glycans was shown by use of a morpholino to the Alg7 gene that encodes the first step in synthesis of the *N*-glycan precursor. We determined whether MBL, as well as galectin-1, agglutinates flagellated *Trichomonas* that are swimming in solution. We tested the effect of topical application of *N*-glycans from the *Saccharomyces mnn1/mnn4* double knockout, which makes *N*-glycans similar to those of *Trichomonas* and HIV, on the recovery of *Tritrichomonas* in the mouse vagina [44].

The second goal was to determine what effect, if any, anti-retroviral lectins designed as topical reagents to prevent heterosexual spread of HIV have on *Trichomonas in vitro* and *Tritrichomonas* in the mouse vaginal model. We used flow cytometry to measure the binding of cyanovirin-N to *Trichomonas* treated with tunicamycin or an Alg7 morpholino, and we determined whether *N*-glycans from the *Saccharomyces mnn1/mnn4* double knockout inhibit binding of cyanovirin-N to parasites. We compared the binding of cyanovirin-N to metronidazole-sensitive and metronidazole–resistant clinical isolates of *Trichomonas* axenized at the Center for Disease Control and Prevention (CDC) [9]. We tested the effects of cyanovirin-N and griffithsin on self-agglutination by *Trichomonas* and on adherence of the parasite to an ectocervical monolayer and to organotypic EpiVaginal tissue cells [45,46]. Finally, we measured the effects of topical application of these anti-retroviral lectins, as well as the 2G12 monoclonal antibody and galectin-1, on recovery of *Tritrichomonas* in the mouse vaginal model.

## Materials and Methods

### Ethics Statement

Culture of *Trichomonas* and *Tritrichomonas* has been approved by the Boston University Institutional Biosafety Committees at Boston University and the University of California-Los Angeles. Mouse infections with *Tritrichomonas* were performed under ABSL-2 protocols (AN-15352) with the approval of the Boston University Institutional Animal Care and Use Committee (BU IACUC). Use of metronidazole-resistant and metronidazole–sensitive *Trichomonas*, which were previously axenized from de-identified clinical isolates at the CDC, has been reviewed by the Boston University Institutional Review Board and judged to not be human subjects research. The organotypic EpiVaginal tissue cells, which derive from de-identified hysterectomy specimens, are a catalog item from MatTek Corporation (Ashland, MA).

### Parasites and cervical/vaginal cell preparations

The genome project G3 strain of *Trichomonas*, the parental strain (B7RC2) for the ricin-resistant mutants, and the two mutants (4–12 and 2E2) have all been described previously [24,47]. The D1 strain of *Tritrichomonas* was received from Lynnette Corbeil of UCSD [2]. Metronidazole-sensitive and metronidazole-resistant *Trichomonas* were axenized (grown without bacteria) from clinical isolates at the CDC [9,48]. Parasites were cultured axenically in TYI-S-33 medium containing 10% bovine serum [49]. Human ectocervical cell line (Ect1 E6/E7), which was a generous gift from Dr. Raina Fichorova of the Brigham and Women’s Hospital, Boston MA, was grown in defined medium, as previously described [24,45]. Organotypic EpiVaginal tissue cells were maintained in DMEM medium containing 10% serum [46].

### Microscopy, flow cytometry, and agglutination assays of lectins bound to the surface of *Trichomonas*

Logarithmic-phase trophozoites were concentrated by low speed centrifugation and washed in chilled phosphate buffered saline (PBS). Cyanovirin-N-labeled trophozoites were washed three times in chilled PBS and then fixed for 10 min at 4°C in 2% paraformaldehyde in 100 mM phosphate, pH 7.4. Cyanovirin-N and griffithsin (1 μg per 100 μl PBS), as well as the anti-2G12 monoclonal antibody made in plants (0.1 μg per 100 μl PBS) (a generous gift of Kevin Whaley of Mapp Biopharmaceutical, San Diego, CA), were labeled with Alexa Fluor dyes and then incubated with intact trophozoites and washed, as described for cyanovirin-N. Recombinant MBL (from Sigma-Aldrich, USA) were also labeled with Alexa Fluor dyes and incubated with intact trophozoites. Nuclei were stained with 0.1 μg/ml DAPI, and organisms were visualized with a DeltaVision deconvoluting microscope (Applied Precision, Issaquah, WA). Images were taken at 100 X primary and deconvolved using Applied Precision’s softWoRx software.

Measurement of the relative amount of cyanovirin-N to *Trichomonas* was performed using a BD FACSCalibur flow cytometer (BD Biosciences, Woburn, MA). The specificity of binding of cyanovirin-N to *N*-glycans was determined in three ways. First, we compared the amount of lectin bound to untreated *Trichomonas* versus *Trichomonas* grown for two days in the presence of 1 μg/ml tunicamycin, which inhibits the phosphoglucosyltransferase (encoded by the Alg7 gene) that catalyzes the first step in *N*-glycan precursor synthesis [26]. Second, trichomonads were electroporated with a morpholino to Alg7 gene (ACTCTTGTTGACATATTTGATTGTT) and then cultured for one day prior to labeling with cyanovirin-N, using methods similar to those for morpholino knockdowns in *Giardia* [50]. Third, a *Saccharomyces mnn1/mmn4* double-knockout was made [44]; yeast were broken and walls were purified; and *N*-glycans were released from yeast walls with PNGaseF. The binding of cyanovirin-N to *Trichomonas* +/- yeast *N*-glycans was then measured using flow cytometry. Similar methods were used to measure binding of recombinant MBL to *Trichomonas*. Each flow experiment was repeated three times.

Agglutination by host MBL and galectin-1 or by anti-retroviral lectins (cyanovirin-N and griffithsin) was performed by incubating log-phase *Trichomonas* (12 X 10^4^ parasites in 200 μl of TYM medium without serum and antibiotics) with 2.5 μg of lectin for 60 min at 37°C. In this assay, control *Trichomonas* incubated without lectin (control group) are flagellated and swimming, so that they are evenly distributed between four equal 50 μl volumes collected from top to bottom of the tubes. In contrast, *Trichomonas* agglutinated by the lectins precipitate to the bottom of the tube. The experiment was performed in duplicate and repeated twice.

### Adherence of *Trichomonas* to ectocervical cells and to organotypic EpiVaginal tissue cells

For ectocervical cell assays, parental strain and ricin-resistant *Trichomonas* mutants were labeled with 10 μM CellTracker Blue CMAC (Invitrogen) and incubated with no lectin or with cyanovirin-N or 1 μg/ml griffithsin (each 1 μg per 100 μl PBS). Parasites (~100,000 per 500 μl PBS) +/- lectins were allowed to adhere to ~300,000 ectocervical cells grown to confluence on a coverslip in a 24 well plate [24]. After 30 min at 37°C coverslips were washed x 4 in PBS to remove nonadherent parasites, fixed in 4% paraformaldehyde, and mounted on slides. Fluorescenated *Trichomonas* present in fifteen 20X fields were counted using Scion Image for Windows. Each treatment was performed in triplicate, and each experiment was performed at least three independent times.

For organotypic EpiVaginal tissue cell assays, logarithmic phase parental strain and ricin-resistant *Trichomonas* mutants were labeled with 10 μM CellTracker green (CMFDA) for 5 min at RT (Invitrogen). CMFDA is concentrated in cytoplasmic vesicles of *Trichomonas*. Some *Trichomonas* trophozoites were also labeled with cyanovirin-N conjugated to Alexa Fluor 594 (1 μg per 100 μl PBS). Trichomonads were washed, resuspended in culture medium without serum, and incubated with EpiVaginal tissue cells for 60 min at 37°C under anaerobic conditions. Three washes in PBS were used to remove unbound parasites from EpiVaginal tissue cells, which were fixed in 2% paraformaldehyde, mounted on a two-chambered cover glass system (Thermo Fisher Scientific, Waltham MA), and observed with the deconvoluting microscope.

### Infections of mice with *Tritrichomonas*

Six weeks old female BALB/c mice were purchased from Taconic (Hudson, NY). Five mice per treatment were infected vaginally with 500 parasites in 10 μl medium +/- 5 μg cyanovirin-N, griffithsin, 2G12 monoclonal antibody, or galectin-1 [2]. Alternatively, *Tritrichomonas* was infected vaginally with *N*-glycans released from *Saccharomyces mnn1/mmn4* double-knockout. After two days 100 μl PBS was used to wash out *Tritrichomonas* parasites, which were incubated overnight in TYM medium to release organisms from viscous vaginal secretions. Parasites were fixed and counted. Each experiment was repeated at least three times.

## Results

### Effects of host mannose-binding lectin and yeast *N*-glycans on *Trichomonas in vitro* and *Tritrichomonas* in mice

**Fig 1:**
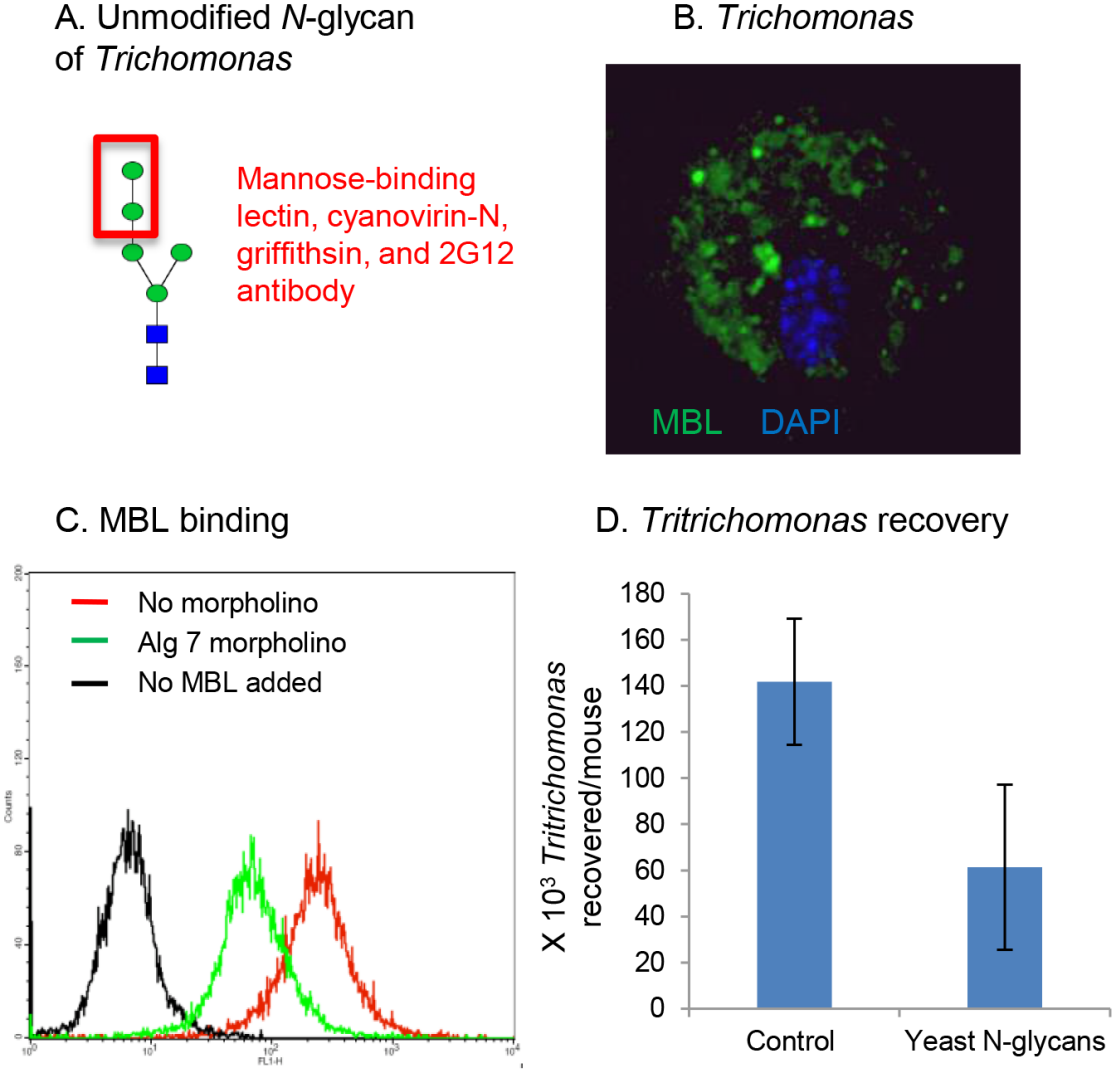
Mannose-binding lectin (MBL) binds to unprocessed *N*-glycans on the surface of *Trichomonas*. A. MBL, as well as anti-retroviral lectins (cyanovirin-N and griffithsin) and 2G12 monoclonal antibody, binds to the single mannose arm (red box) of an unprocessed *N*-glycan of *Trichomonas*. B. MBL (green) labels the surface of *Trichomonas*. The nucleus is labeled with DAPI. C. Flow cytometry shows that labeling with the MBL (red) is decreased when *Trichomonas* is treated with an Alg7 morpholino (green) that decreases synthesis of the *N*-glycan precursor. Unlabeled parasites are shown in black. D. *N*-glycans from the *Saccharomyces mnn1/mmn4* double-knockout decrease by 40% the recovery after two days of *Tritrichomonas* in the vagina of mice (average of three experiments with five mice each).

**Fig 2:**
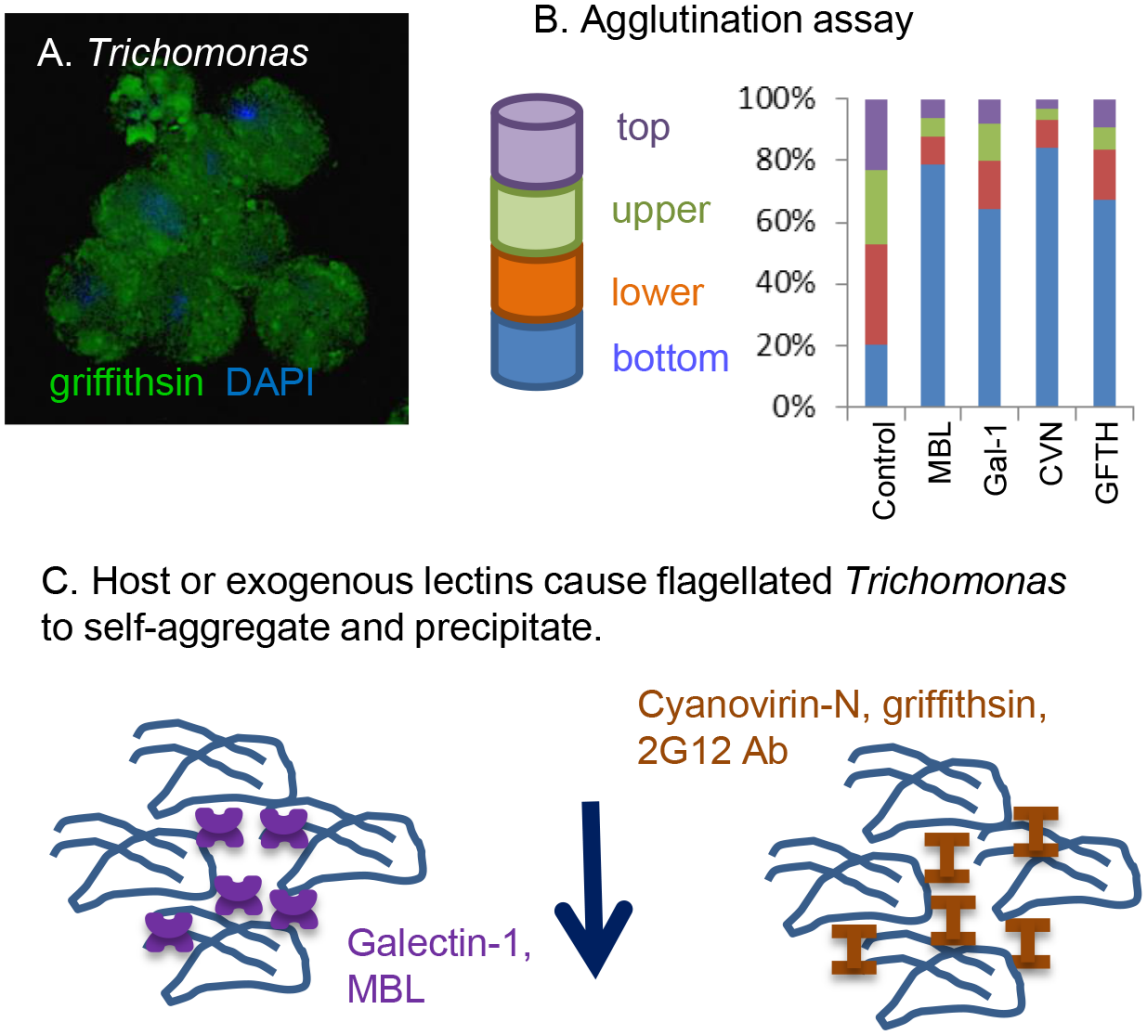
Host lectins (galectin-1 and MBL), as well as anti-retroviral lectins (cyanovirin-N and griffithsin) and 2G12 monoclonal antibody, agglutinate flagellated *Trichomonas* and cause them to precipitate. A. Fluorescence micrograph of *Trichomonas* aggregated by griffithsin (green). Nuclei are labeled with DAPI. B. In the absence of host or exogenous lectins added to TYM medium (control group), flagellated *Trichomonas* parasites swim and remain in roughly equal numbers in the top (purple), middle (green and rust), and bottom (blue) 50 μl fractions of a 200 μl suspension. In contrast, host MBL and galectin-1 (Gal-1), as well as exogenous cyanovirin-N (CVN) and griffithsin (GFTH), agglutinate swimming *Trichomonas* and cause them to precipitate, so that the vast majority of the parasites are in the bottom 50 μl fraction (blue). The average of two experiments, each performed in duplicate, with very similar results is shown. C. Model shows that host or exogenous lectins agglutinate flagellated *Trichomonas* and cause them to precipitate (blue arrow).

**Fig 3:**
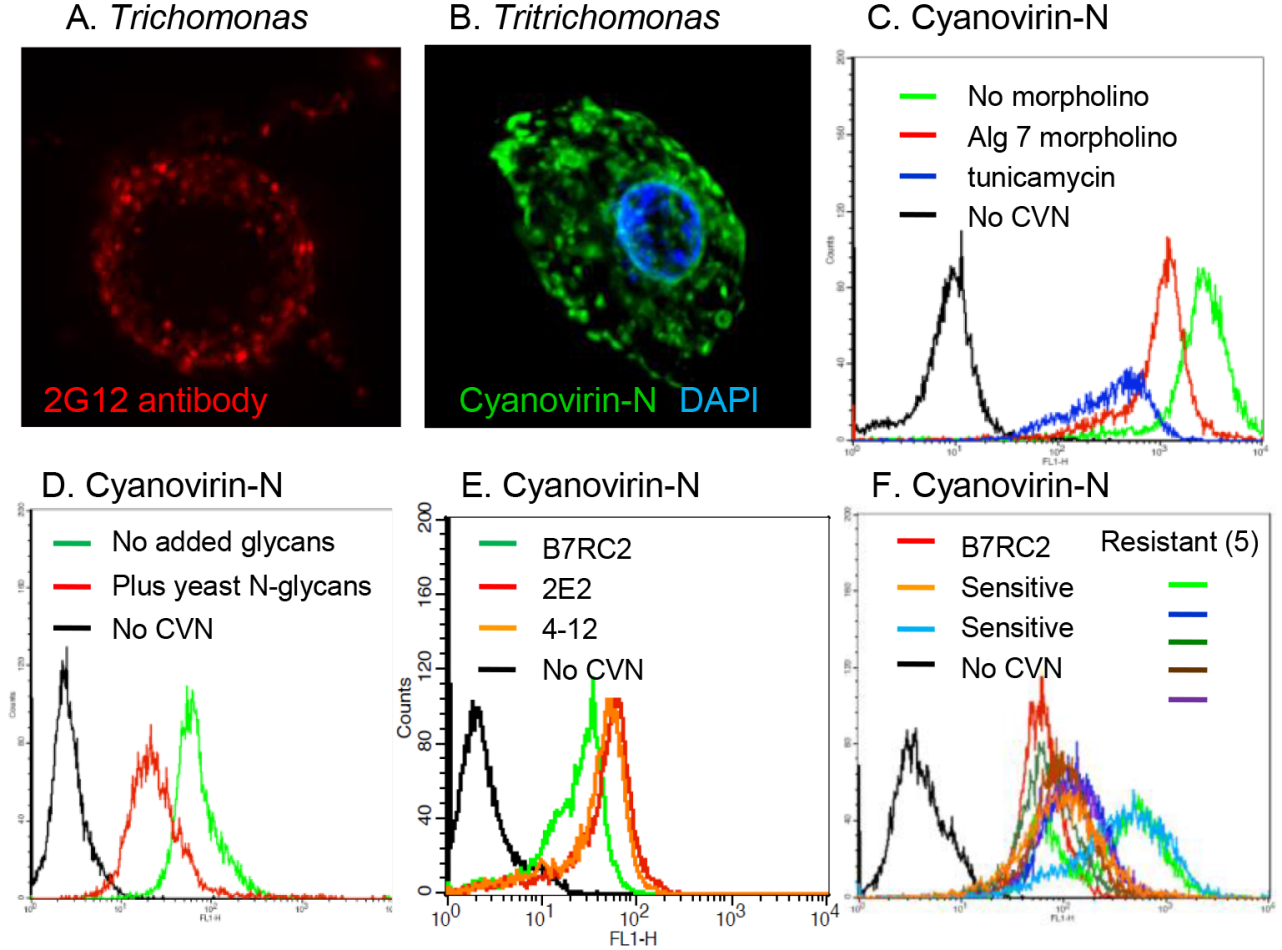
Anti-retroviral lectins and the 2G12 monoclonal antibody bind *N*-glycans of *Trichomonas*. A. Fluorescence micrographs show binding of griffithsin 2G12 antibody (red) to the surface of *Trichomonas*. B. Fluorescence micrograph shows binding of cyanovirin-N to the surface of *Tritrichomonas*. C. Flow cytometry shows that labeling with cyanovirin-N (green) is decreased when *Trichomonas* is treated with an Alg7 morpholino (red) or treated with tunicamycin (blue), both of which inhibit the first step in synthesis of the *N*-glycan precursor. D. Flow cytometry shows decreased binding of cyanovirin-N to *Trichomonas* (green) in the presence of *N*-glycans from the *Saccharomyces mnn1/mmn4* double-knockout (red). E. Flow cytometry shows binding of cyanovirin-N to ricin resistant mutants (2E2 and 4–12) is slightly greater than binding to parent strain (B7RC2). F. Flow cytometry shows that binding of cyanovirin-N to two metronidazole-sensitive clinical isolates (bright blue and tan) and five metronidazole-resistant clinical isolates (green, dark blue, light blue, brown, and purple) from the CDC is as great or greater than binding of cyanovirin-N to B7RC2 (red), the parent strain for the ricin-resistant mutants.

**Fig 4:**
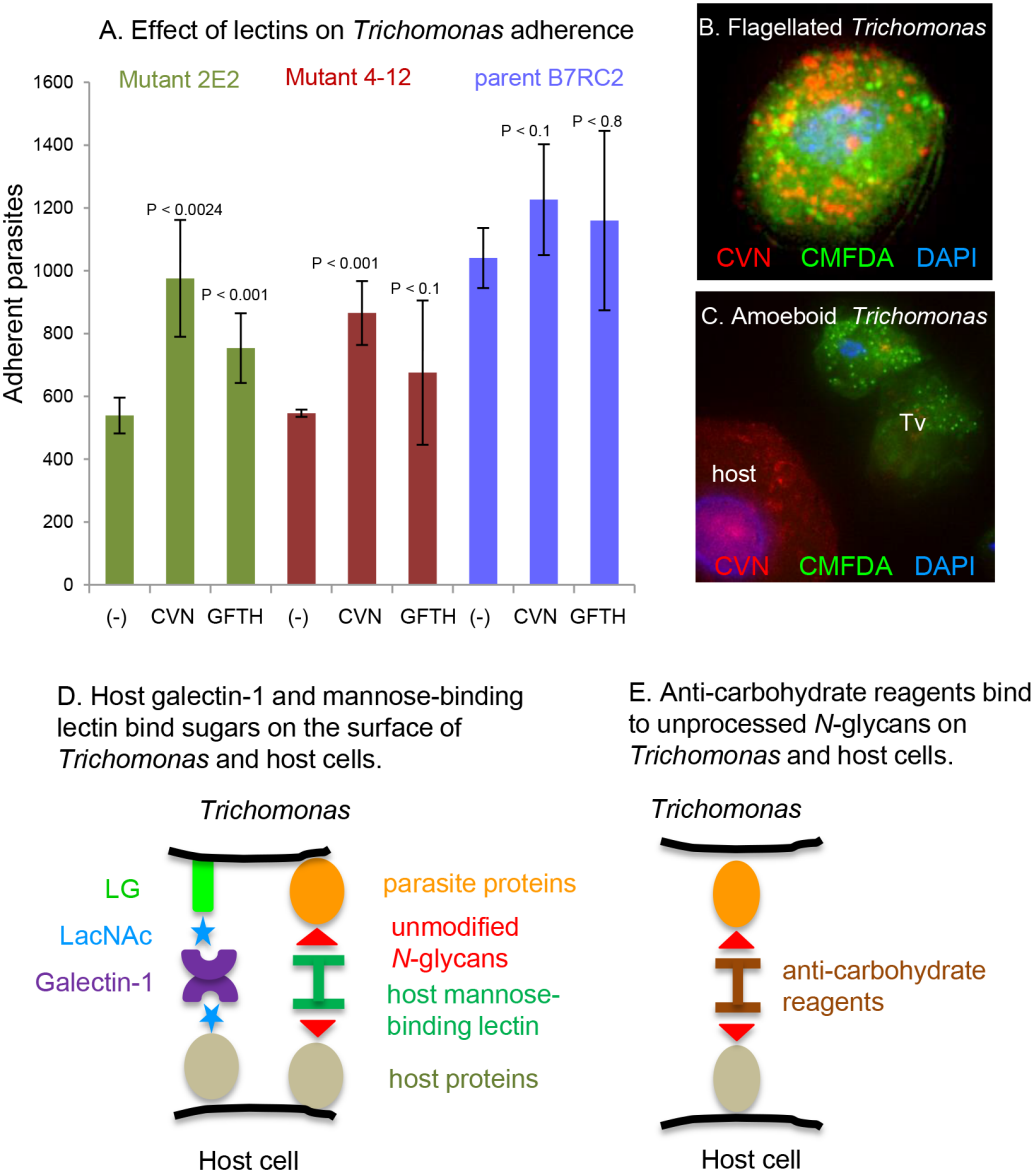
Anti-retroviral lectins increase the adherence of ricin-resistant *Trichomonas* mutants to ectocervical cells, while cyanovirin-N is lost from the parasite surface when *Trichomonas* binds to EpiVaginal tissue cells and converts into an ameboid form. A. Cyanovirin-N (CVN) increases the adherence of ricin-resistant mutant *Trichomonas* (2E2 and 4–12) to ectocervical cells *in vitro*, while griffithsin (GRFT) has less effect. Neither lectin has much effect on the parent B7RC2 strain, which is much more adherent to ectocervical cells in the absence of lectin. Data is average of three experiments. B. A flagellated *Trichomonas* labeled green with CMFDA (green), which concentrates in secretory vesicles, has cyanovirin-N (red) on its surface. C. When CMFDA-labeled *Trichomonas* adhere to organotypic EpiVaginal tissue cells, most of the cyanovirin-N is lost. Note that the host EpiVaginal cell (lower left) binds cyanovirin-N. D. A model for how host lectins affect adherence of *Trichomonas* to host cells is based upon experiments here and previously published experiments [24]. Galectin-1 (purple) secreted by the host epithelial cells cross-links LacNAc residues (bright blue) present on glycans of host proteins (grey) and on parasite LG (bright green). Similarly, host mannose-binding lectin (deep green) cross-links unmodified *N*-glycans (red) present on host and parasite proteins. E. Exogenous anti-retroviral lectins (brown) cross-link *N*-glycans on the surface of *Trichomonas* and host cells.

Our hypothesis here is that host MBL, which binds to unmodified *N*-glycans of HIV, will also bind to unmodified *N*-glycans on *Trichomonas* (Fig 1A) [33–36]. In support of this idea, we found that recombinant MBL binds to the surface of *Trichomonas* (Fig 1B). The non-homogeneous binding of MBL (and of anti-retroviral lectins in Figs 2A, 3A, 3B and 4B) is likely subsequent to the ridged and ruffled surface of trichomonads labeled in solution. The specificity of the binding of MBL to unmodified *Trichomonas N*-glycans was shown by decreased binding of MBL to *Trichomonas* pre-treated with an Alg7 morpholino, which decreases expression of the enzyme that is the first step in *N*-glycan precursor synthesis (Fig 1C) [26]. Trichomonads, which are flagellated and swim as individuals in solution, are agglutinated by MBL and sink to the bottom of a test tube (Fig 2B and 2C). These aggregates resemble “swarms” of *Trichomonas* formed in response to stress [51]. Similarly, recombinant galectin-1, which binds to the surface of *Trichomonas* [22], also causes flagellated trichomonads to self-aggregate and precipitate.

Because *Trichomonas* weakly infects mice while *Tritrichomonas* makes a robust infection that ascends into the uterus, *Tritrichomonas* was used to explore the possible role for parasite *N*-glycans in pathogenesis [2,3]. *Tritrichomonas* was directly applied to the mouse vagina +/- *N*-glycans from a *Saccharomyces mnn1/mnn4* double knockout, which makes *N*-glycans similar to those of *Trichomonas* and HIV [44]. These yeast *N*-glycans, which have been used to raise antibodies targeted to the *N*-glycans present on gp120 of HIV, decreased recovery of *Tritrichomonas* by 40% (P < 0.001) after two days in the mouse vaginal model (Fig 1D). Because we did not perform a histological examination of the vagina nor are we detecting luciferase expressing parasites by bioluminescence, we cannot rule out that some adherent parasites remain after the vaginal wash. Alternatively, some *Tritrichomonas* may have migrated to the uterus and were not counted.

### Anti-retroviral lectins and the 2G12 monoclonal antibody bind *N*-glycans of *Trichomonas*.

The anti-retroviral lectins cyanovirin-N and griffithsin and the anti-carbohydrate 2G12 monoclonal antibody bind to the surface of *Trichomonas* and *Tritrichomonas* (Figs 2A, 3A, 3B and 4B) [37–40]. The specificity of cyanovirin-N for *Trichomonas N*-glycans was shown in two ways [30]. First, treatment of *Trichomonas* with an Alg7 morpholino or with tunicamycin, both of which target the first enzyme in *N*-glycan precursor synthesis, decreases binding of cyanovirin-N to the parasites, as measured by flow cytometry (Fig 3C) [26,50]. Second, binding of cyanovirin-N is inhibited by co-incubation with *N*-glycans released from a *Saccharomyces mnn1/mmn4* double-knockout (Fig 3D) [44]. As a control for the adherence experiments (see below), we showed that binding of cyanovirin-N is slightly greater to ricin-resistant mutants (2E2 and 4–12) than to the parent strain (B7RC2) (Fig 3E) [24].

We tested the binding of cyanovirin-N to axenized clinical isolates of *Trichomonas* from the CDC, which are either sensitive to metronidazole (two isolates) or resistant to metronidazole (five isolates) [9,48]. All but one of the clinical isolates binds cyanovirin-N better than the parent strain (B7RC2) for the ricin-resistant mutants (Fig 3F). The two clinical isolates that bind cyanovirin-N best include one isolate that is metronidazole-sensitive and one that is metronidazole-resistant. These results suggest *N*-glycan synthesis and metronidazole activation are unrelated, and so anti-retroviral lectins might be used as an additional, local treatment for metronidazole-resistant *Trichomonas* or for pregnant women, who cannot take tinidazole.

### Effects of anti-retroviral lectins on *Trichomonas in vitro* and *Tritrichomonas* in mice

Like MBL and galectin-1, anti-retroviral reagents (cyanovirin-N, griffithsin, and the 2G12 monoclonal antibody) each cause flagellated trichomonads to self-aggregate and precipitate (Fig 2A and 2B). Previous studies have shown that ricin-resistant mutants of *Trichomonas* adhere less well than the parental strain to ectocervical cells *in vitro* and that galectin-1 increases adherence [22,24]. Here we found that cyanovirin-N nearly doubles the adherence of ricin-resistant mutants to ectocervical cells, while griffithsin has a more modest effect on adherence (Fig 4A). In contrast, neither of the anti-retroviral lectins have much effect on adherence of the parent strain (B7RC2), which adheres well to ectocervical cell monolayers in the absence of lectins.

Close examination of *Trichomonas* adhering to organotypic EpiVaginal tissue cells, which form a multilayered squamous epithelium that closely resembles that of the vagina [46], showed three findings. First, cyanovirin-N increases adherence to EpiVaginal cells of ricin-resistant mutants, as was shown with ectocervical cell monolayers (data not shown). Second, cyanovirin-N binds well to EpiVaginal cells, which have unprocessed *N*-glycans on their surface in addition to complex *N*-glycans that do not bind cyanovirin-N (Fig 4C). Third, when trichomonads adhere to the EpiVaginal cells and transform from a flagellated to an ameboid form, they shed most of the cyanovirin-N that is present on swimming parasites (Fig 4B and 4C). Although cyanovirin-N agglutinates flagellated parasites and causes them to sink to the bottom of a tube or flask (Fig 2B and 2C), cyanovirin-N and the other lectins that bind to *Trichomonas* do not cause them to convert to an ameboid form, which only occurs when trichomonads adhere to host cells or to a derivatized surface [13–18,22,24,25].

**Fig 5:**
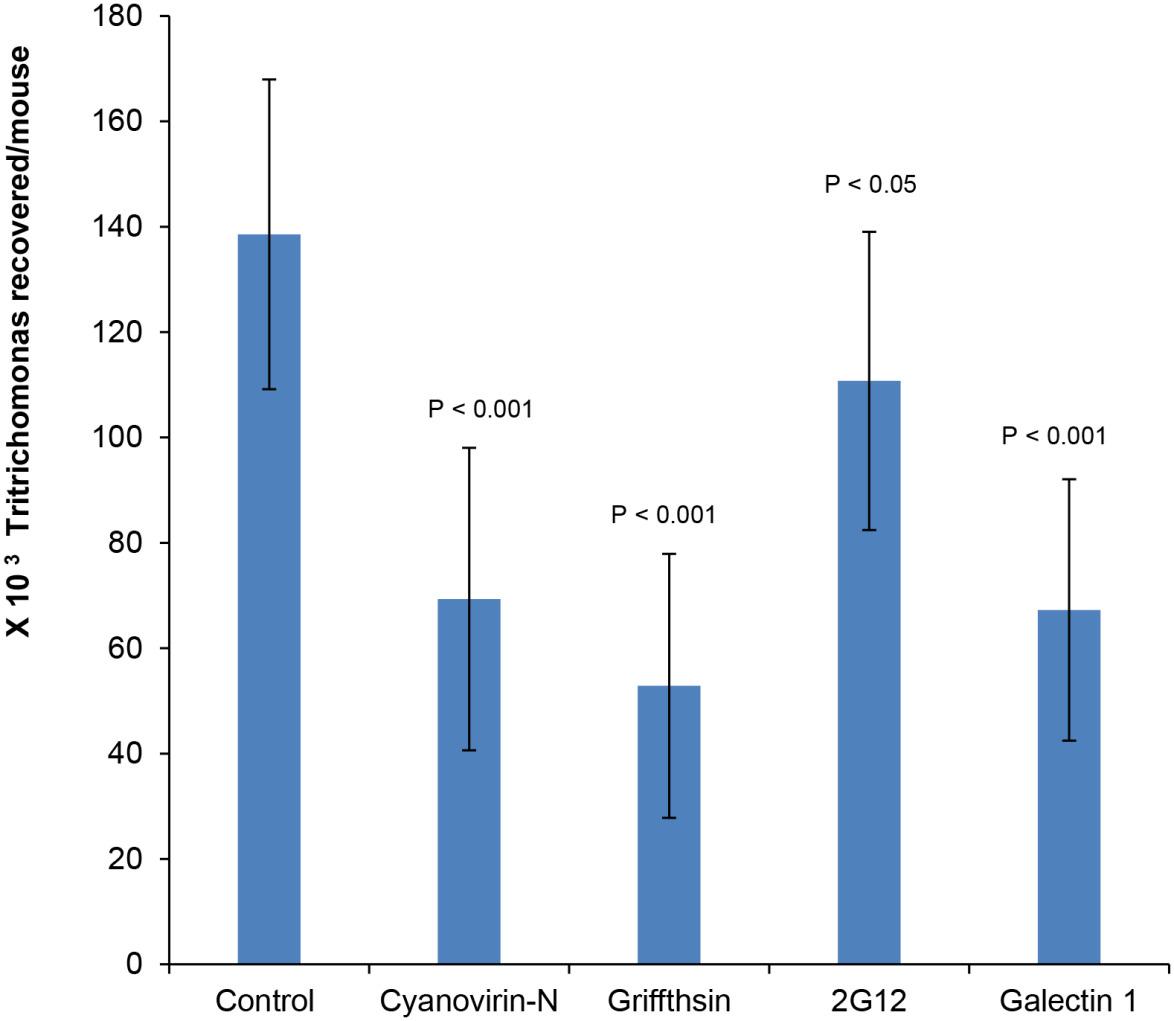
Anti-retroviral lectins decrease recovery of *Tritrichomonas* in a mouse vaginal model. Recovery of *Tritrichomonas* after two days in the vagina of mice shows a modest but statistically significant decrease by co-infection with cyanovirin-N (63% decrease), griffithsin (70% decrease), or galectin-1 (51% decrease). In contrast, there is little effect of the 2G12 monoclonal antibody. Data is the average of three experiments each containing five mice per treatment.

Topical application of cyanovirin-N or griffithsin at the time of infection with *Tritrichomonas* reduced the recovery of parasites from the vagina of mice after two days by 63% or 70%, respectively (Fig 5). Although the 2G12 monoclonal antibody had little effect on the recovery of *Tritrichomonas* from the mouse vagina, galectin-1 reduced recovery by 51% (Fig 5). These results, which have the same caveats as experiments with the yeast *N*-glycans with regards to missing adherent parasites or those that have migrated to the uterus, suggest anti-retroviral lectins have a modest potential for treating or preventing human infections with *Trichomonas*.

## Discussion

### Revised models for *Trichomonas* adherence and pathogenesis

Previous studies showed that adherence of *Trichomonas* to ectocervical cells is mediated in part by host galectin-1, which is bivalent and binds to LacNAc on parasite LG and to LacNAc on host cells (Fig 4D) [22–24]. The present experiments show that galectin-1 agglutinates flagellated *Trichomonas*, so they are less able to swim and divide (Fig 2B and 2C). In contrast, galectin-1 decreases recovery of *Tritrichomonas* in the mouse vaginal model (Fig 5). This discrepancy, which is also the case for the anti-retroviral lectins, suggests the importance of the *in vivo* model, even if it is with the bovine rather than the human parasite and even if there is a possibility that all parasites have not been obtained in the washes [2,3].

MBL, another important player in the host innate immune response, binds to unmodified *N*-glycans on the surface of *Trichomonas* and causes parasites to self-aggregate (Figs 1A–1C, 2B and 2C) [33–36]. Whether complement activation by MBL plays a role in *Trichomonas* infections was not examined. Recovery of *Tritrichomonas* in the mouse vaginal model is decreased in the presence of *N*-glycans from the *Saccharomyces mnn1/mmn4* double-knockout (Fig 1D) [44]. This result suggests roles for *N*-glycans of the host and/or parasite in colonization of trichomonads *in vivo*.

The results here, as well as recent experiments that demonstrate roles for parasite exosomes, bacterial vaginosis, and host cytokines [7,8,13–15,22–24,52], suggest that mechanisms of pathogenesis by *Trichomonas* are multiple and complex. Similarly, how anti-retroviral lectins reduce recovery of *Tritrichomonas in vivo* (next section) is likely more complicated than how these same reagents block invasion of HIV invasion into host cells [37–40,53].

### Modest potential for anti-retroviral lectins to prevent *Trichomonas* infections

The logic for testing anti-retroviral lectins versus *Trichomonas* included the following. Spectrometric studies show that many *Trichomonas* surface and secreted proteins have no known function and are unique to the parasite, so it is difficult to choose vaccine candidates (our unpublished data and [29]). The majority of unique *Trichomonas* proteins are absent from the predicted proteins of *Tritrichomonas* (our unpublished data), so that it is necessary to overexpress the *Trichomonas* vaccine candidate in transformed *Tritrichomonas* and then use these parasites in the mouse model [2,3,54]. In contrast, nearly all *Trichomonas* surface and secreted proteins have *N*-glycan sites. Like HIV and HSV, *Trichomonas* is sexually transmitted, so an anti-retroviral reagent present in the vagina might be used to prevent all three infections [1,6,37–40]. The big advantage of using the anti-retroviral reagents versus *Trichomonas* is that GMP production, pharmacokinetic studies, and toxicity tests, all of which are likely prohibitively expensive for a new anti-trichomonad reagent, will have already been performed in an effort to prevent heterosexual transmission of HIV [39,40]. Finally, the use of a topical anti-retroviral reagent along with systemic treatment with tinidazole or metronidazole to treat drug-resistant *Trichomonas* is similar to the idea of combining anti-retroviral lectins with conventional anti-retroviral drugs to target HIV [55].

Similar to host galectin-1 [16], anti-retroviral lectins and 2G12 antibody increase adherence of trichomonads to ectocervical cells *in vitro* (Fig 4A). This effect is most likely by cross-linking unprocessed *N*-glycans on the surface of parasite and host (Figs 3B–3F and 4E). Agglutination of flagellated parasites by the anti-retroviral lectins may also contribute to their effect on adherence *in vitro* and colonization *in vivo* (Fig 2A–2C). While anti-retroviral lectins have been shown to decrease infectivity of numerous viruses in animal models, this is the first time that these lectins have been tested versus a parasite in a mouse model (Fig 5) [41–43]. The modest reduction in *Tritrichomonas* recovered in the presence of anti-retroviral lectin (with many caveats about the counting) is much less than the total elimination of parasites that can be accomplished with metronidazole or tinidazole [9]. Indeed the partial reduction in parasite recovery is more similar to that seen by vaccination against *Trichomonas*, *Entamoeba*, or *Giardia* [10,56,57]. An alternative conclusion from the studies here is that the use of anti-retroviral reagents to prevent heterosexual transmission of HIV and HSV is unlikely to have a large negative effect on *Trichomonas* infections. Because *Trichomonas* does not infect easily infect mice, anti-retroviral lectins might be tested in an excellent but expensive pigtail macaque model of *Trichomonas* infection +/- HIV [58–60].
